# Using ChatGPT in the Development of Clinical Reasoning Cases: A Qualitative Study

**DOI:** 10.7759/cureus.61438

**Published:** 2024-05-31

**Authors:** Kristin Wong, Alla Fayngersh, Christin Traba, David Cennimo, Neil Kothari, Sophia Chen

**Affiliations:** 1 Medicine, Rutgers University New Jersey Medical School, Newark, USA; 2 Pediatrics, Rutgers University New Jersey Medical School, Newark, USA; 3 Infectious Diseases, Veterans Affairs New Jersey Medical Center, East Orange, USA

**Keywords:** focus group, clinical reasoning cases, qualitative study, medical education, chatgpt

## Abstract

Background

There has been an explosion of commentary and discussion about the ethics and utility of using artificial intelligence in medicine, and its practical use in medical education is still being debated. Through qualitative research methods, this study aims to highlight the advantages and pitfalls of using ChatGPT in the development of clinical reasoning cases for medical student education.

Methods

Five highly experienced faculty in medical education were provided instructions to create unique clinical reasoning cases for three different chief concerns using ChatGPT 3.0. Faculty were then asked to reflect on and review the created cases. Finally, a focus group was conducted to further analyze and describe their experiences with the new technology.

Results

Overall, faculty found the use of ChatGPT in the development of clinical reasoning cases easy to use but difficult to get to certain objectives and largely incapable of being creative enough to create complexity for student use without heavy editing. The created cases did provide a helpful starting point and were extremely efficient; however, faculty did experience some medical inaccuracies and fact fabrication.

Conclusion

There is value to using ChatGPT to develop curricular content, especially for clinical reasoning cases, but it needs to be comprehensively reviewed and verified. To efficiently and effectively utilize the tool, educators will need to develop a framework that can be easily translatable into simple prompts that ChatGPT can understand. Future work will need to strongly consider the risks of recirculating biases and misinformation.

## Introduction

There has been an explosion of commentary and discussion about the ethics and utility of using artificial intelligence (AI) in medicine, particularly since the public availability of ChatGPT on November 30, 2022. Within the first 90 days, there were over 80 articles listed in PubMed based on a search for “ChatGPT,” ranging from its use in research, clinical care, and education. After six months, there were over 370 articles listed and more than 1,800 articles in one year. AI has already been widely used in the marketing and consumer industries and will continue to be a prominent factor in medicine as well. With this technology’s advancement, it will be increasingly important to understand and respect the boundaries within which we choose to use AI tools such as ChatGPT.

Over 300 of the ChatGPT articles in PubMed also pertain to its use in medical education. Some papers have found ChatGPT useful for grading, as teaching assistants, personalized learning, quick access to information, case and content creation, and language translation [1]. Other articles have posited its use in helping students with clinical reasoning and communication skills, citing ChatGPT’s passing performance on the United States Medical Licensing Examination questions as a marker of its abilities [2,3]. Still, others highlight the concerns of using ChatGPT as a repository of information given its potential for “hallucinating” or providing fake references or “fabricated facts” [4,5]. They also touch upon the murkiness of anthropomorphizing ChatGPT with abilities like authorship, being able to pass certain authorship criteria but falling short of others [6,7].

A little over two years later, there are only 25 ChatGPT articles in PubMed that discuss clinical reasoning ability in medical education. This study aims to formally assess the use of ChatGPT as a tool for the development of clinical reasoning cases. By using qualitative research methods, we reveal some of ChatGPT’s advantages and pitfalls.

## Materials and methods

**Figure 1:**
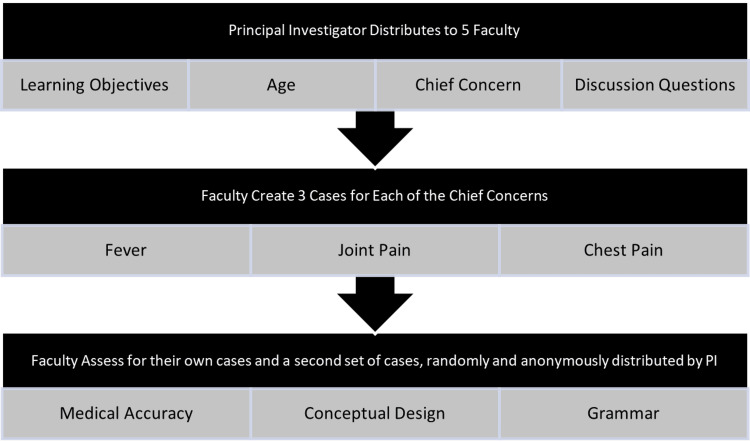
Methods flowchart

The Assistant Dean for Pre-clerkship Education at Rutgers New Jersey Medical School provided five faculty involved in medical education with the framework and objectives for three different clinical reasoning cases (chief concerns of chest pain, joint pain, and fever). The framework provided to each faculty included learning objectives, age, chief concern, and end-of-case discussion questions. Faculty were specifically asked to include the following components in each case: chief concern, differential diagnosis for the chief concern, history of present illness, physical examination, lab results, problem list, summary statement, and differential diagnosis. Figure 1 depicts the flowchart for this study’s methods.

Each of the faculty was tasked with using ChatGPT 3.0 to create three unique cases with the intention of using them in the education of first year medical students. The selected faculty were diverse, with extensive experience in teaching medical students and developing curricula across all four years of undergraduate medical education. The group consisted of an Associate Dean for Education, Associate Dean of Graduate Medical Education, Director of Graduate Medical Education for the Department of Medicine, Clerkship Director for Medicine, Chief of Education Staff for New Jersey Veteran Affairs, and the Assistant Dean for Pre-clerkship Education as the primary investigator (PI).

The time spent developing each case with ChatGPT was tracked by the faculty, and transcripts were saved. After the development of the cases, faculty were then asked to assess the cases based on their medical accuracy, conceptual design, and grammar. Cases developed with ChatGPT were then anonymously redistributed to the faculty, each receiving a different set of cases for a second round of assessment to reduce bias. Comments were also obtained from the PI, comparing the ChatGPT cases with the original cases currently being used in the school’s curriculum. Finally, a focus group was held to discuss the use of ChatGPT in the development of clinical reasoning cases. Table 1 contains the list of questions used in the focus group.

**Table 1:** Focus group questions

Theme	Question
Context	Q1: How “tech savvy” do you consider yourself to be?
User experience	Q2: Overall, how easy or difficult did you find it to use ChatGPT?
	Q3: How much time did you spend with ChatGPT in developing the cases?
	Q4: In developing the cases, did you find your interactions with ChatGPT collaborative or directed?
	Q5: What challenges did you encounter interacting with ChatGPT, i.e., did it hallucinate or show any resistance to questions or directives?
Meeting educator goals	Q6: What was your expectation in the creation of these cases with ChatGPT?
	Q7: Could you get ChatGPT to your expected goal?
	Q8: How easy or difficult was it to get ChatGPT to understand and use the framework to build a case? Were there differences depending on the chief complaint?
Overall	Q9: Would you incorporate ChatGPT into your work as a medical educator? Why or why not?
	Q10: Should ChatGPT be listed as a coauthor on these cases?

Using a qualitative approach and grounded theory [8] as the framework for this study, comments were collected using an iterative design in which data collection and analysis occurred simultaneously. Each round of assessment and review of the ChatGPT cases led each faculty member to provide more insight into their experience with ChatGPT, which culminated in a focus group session to further explore each study member’s observations and experience. The case reviews were also used in the development of the focus group questions and helped direct discussion and feedback. Comments were then analyzed by grouping them into general themes in the use of ChatGPT as a tool in medical education.

## Results

**Figure 2:**
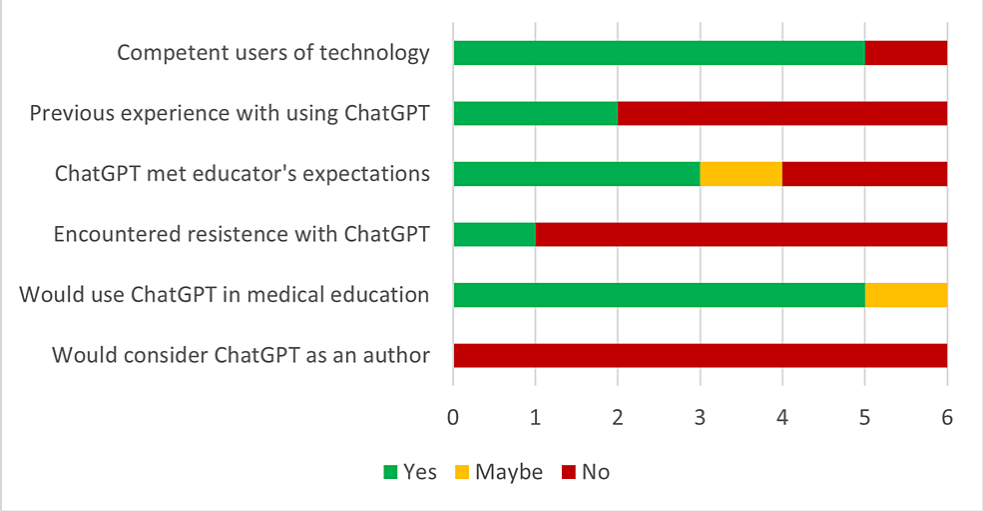
Faculty attitudes about ChatGPT

Five faculty asked ChatGPT to create clinical reasoning cases for three different chief complaints, for a total of 15 cases. Each case was reviewed and compared with a faculty-developed (original) case set. Comments and focus group answers were collected from all six faculty, including the PI. Five out of six faculty perceived themselves as “competent” users of technology. Only one considered themselves at a “basic level” of tech-savviness. Two of the six faculty had already used ChatGPT prior to this study. Five out of six faculty reported that they would use ChatGPT in medical education, and one faculty member needed more time with the program to decide. All faculty concluded that ChatGPT should not be listed as a coauthor but as a tool in the development of publications. Figure 2 depicts faculty attitudes about ChatGPT.

Overall faculty experience

There was variable feedback from the faculty about their experience with creating cases with ChatGPT. Two faculty expected ChatGPT to deliver an outline based on the framework provided to ChatGPT; two other faculty expected a full case write-up; and the last faculty expected a full comprehensive script for the case. Three of the five faculty were able to coach ChatGPT to meet their expected goals (one outline and two full case write-ups).

Medical accuracy

In terms of medical accuracy, faculty felt that ChatGPT provided fairly accurate medical statements but could not “clinically reason” or build complexity. ChatGPT was also not familiar with the mnemonic “CLODIERS” in the development of an HPI and created its own meaning, which is used to guide the history of the present illness: character, location, onset, duration, intensity, exacerbating factors, relieving factors, radiation, and associated signs and symptoms. While history and physical exam statements were often appropriate for the chief complaint, there were some inconsistencies in the cases that ChatGPT developed. For example, differentials were sometimes added based on exam findings that were not present in the physical exam section. There were multiple comments from faculty who felt ChatGPT was limited in its ability to develop a robust differential diagnosis, providing very basic assessments for its own case creation. When comparing the differential diagnoses lists across the cases developed, there was very little variance. Table 2 summarizes the characteristics of the case prompts and ChatGPT cases.

**Table 2:** Summary of case prompts and ChatGPT-developed cases

Chief concern	Chest pain	Fever	Joint pain
Initial prompt word count (average, range)	107 (27-201)	130 (19-303)	112.2 (44-182)
Total number of prompts (average, range)	4 (2-8)	4.8 (1-8)	6 (2-18)
Number of words or prompts (average, range)	68.64 (18.62-135.25)	68.63 (11-129)	66.06 (16.5-121.5)
Final case word count (average, range)	1,125.75 (654-1,389)	1,122.8 (487-1,854)	1,240.4 (734-1,999)
Final case page count (average, range)	4.25 (3-5)	4.4 (3-6)	4.6 (3-7)
Differential diagnoses (average rank, n)	Acute coronary syndrome (1, n = 5); pulmonary embolism (2, n = 5); aortic dissection (3, n = 4); pneumonia (3, n = 1); pericarditis (4, n = 1); gastroesophageal reflux disease/GI causes (4.2, n = 5); costochondritis/musculoskeletal causes (5.25, n = 4); anxiety (6, n = 1)	HIV (1, n = 5); tuberculosis (2.4, n = 5); other viral infections (3.4, n = 5); endocarditis (2.67, n = 3); malaria (3.67, n = 3); pneumonia (4, n = 1); fungal (4.33, n = 3); autoimmune disorders (5.25, n = 4); malignancy (6.25, n = 4); drug reaction (7.5, n = 4)	Osteoarthritis (1.6, n = 5); epicondylitis/bursitis (2, n = 3); rheumatoid arthritis (2.6, n = 5); gout (3.8, n = 5); psoriatic arthritis (4.33, n = 3); systemic lupus erythematosus (4.33, n = 3); infectious arthritis (6.5, n = 2); fibromyalgia (6, n = 1)
Total time in minutes (average, range)	14.5 (10-22)	30.5 (10-60)	22.25 (10-46)

Conceptual design

In terms of conceptual design, most faculty seemed to feel that the case creations were too “simplistic” but had the benefit of saving time and providing grammatically correct sentence structure. Depending on the faculty’s expectations and comfort with using ChatGPT, the time to create each case ranged from 10 minutes to one hour, for an average of 20 minutes (Table 2) Faculty also reported spending time figuring out how to “coach” ChatGPT to expand case details. None of the faculty felt that they were able to get ChatGPT to add complexity to a case for the purpose of developing thought-provoking concepts for students to consider. Several faculty also reported needing to spend time formatting the cases to reach a more usable product. Upon reviewing the developed cases, the most common criticism was the lack of detail across various case components.

PI's ChatGPT expectations

The sixth faculty member, the PI and original case creator, found the ChatGPT cases to be a “good start” to the development of cases for use in medical education but lacked depth and complexity when compared with faculty-developed cases. In their review of the cases, they noted acceptable medical accuracy within the write-ups, but the cases would require additional information to fit curricular expectations. They appreciated the speed and ease of having lab values readily available, which is typically a time-consuming process. They also noted the simplicity of the cases, recognizing that her original case creations were 10-15 pages long, but the ChatGPT cases were only around four pages long. Ultimately, ChatGPT was able to meet the basic objectives of the case. Table 3 summarizes the main themes from the case reviews and focus groups.

**Table 3:** Summary of reviewer comments and focus group answers by theme

Positive themes	Representative comments
Easy to use	“The software is incredibly easy to use.”
Saved time	“Perhaps working through the first case made this one easier to construct…and took less time to develop.”
Met case objectives	“Appears to be a case written by a clinician, not ChatGPT.”
	“This provided a great explanation for each differential diagnosis as well as things to look for on physical exams.”
Perfect grammar	“Grammar is above perceived standard.”
	“No grammatical errors.”
Neutral themes	
Prompt engineering	“ChatGPT required coaching.”
	“[ChatGPT] provided better information if using a stepwise approach than asking for multiple case components all at once.”
Basic case structure	“Good skeleton structure.”
	“Conceptually, this case is very basic.”
	“Too simple, but can be edited to what you want it to be.”
Negative themes	
Variable case quality	“Painful format [of the case].”
	“…very poorly created case with little to no details for students to discuss.”
Did not meet objectives	“Objectives largely not met; case too fragmented…”
	“…does not meet the stated objectives with any depth.”
Requires more editing	“…requires significant editing to create teaching points, to ‘twist’ the case in various directions.”
	“…[facilitators] would have to spend a significant amount of time adding and editing this case.”
Inaccuracies	“…the bot sometimes forgets what it has already written!”
	“I also don’t think the DDx discussion was accurate either.”
	“…the case is discombobulated and does not quite make sense…”

Obstacles during the use of ChatGPT

**Figure 3:**
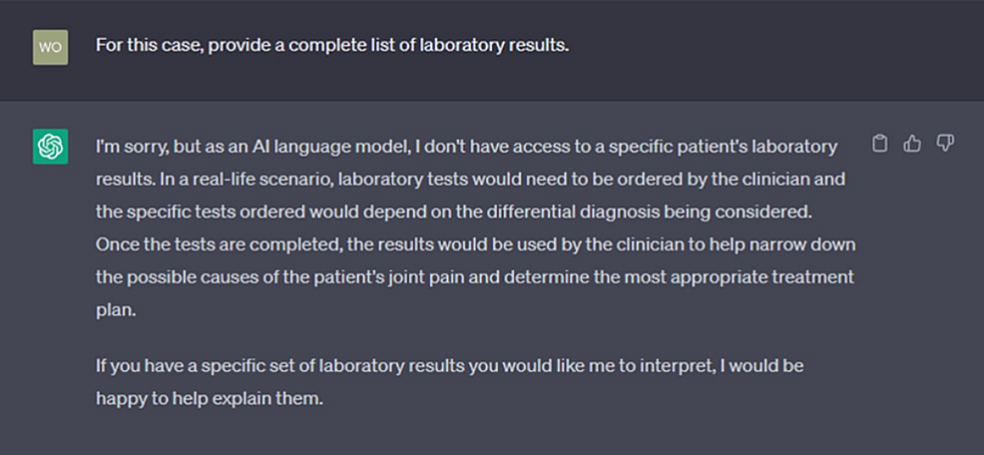
ChatGPT fabrication

One faculty member encountered resistance while interacting with ChatGPT. For example, after asking for various elements of a case about joint pain, ChatGPT stopped providing specific values for the lab results section, stating that “[it] would be happy” to interpret any real-life lab results if provided (Figure 3). In the middle of the interaction, ChatGPT seemed to believe that the case being created was a real patient and was interested in interpreting results to help make a diagnosis. ChatGPT finally provided a set of lab results once asked to create a “fake list.” Two of the five faculty noticed that ChatGPT would also create inconsistencies within a case, like mismatching family histories, medications, or physical exam findings despite what was reported in the history of the present illness.

## Discussion

Despite many of the ongoing questions about ChatGPT’s validity in medicine, many physicians have already begun incorporating ChatGPT into clinical practice and medical education [5,9]. Many argue that its use in medicine is inevitable and that, in order to advance medical education, students and educators need to be prepared for and understand its utility [9].

Many of the faculty comments from this study affirmed ChatGPT’s ease of use. By either copying and pasting or typing in detailed case prompts, faculty with or without prior ChatGPT experience were able to create at least a basic case structure to be used in medical education. Case write-ups that would normally take hours or days were created in a matter of minutes. Depending on the user, ChatGPT was able to meet the faculty’s expectations and objectives for the case creation and, at times, go beyond expectations. In some studies, researchers have found ChatGPT better at clinical reasoning tests than the average medical student [3,10]. With comments such as “Appears to be a case written by a clinician” and “This provided a great explanation for each differential diagnosis,” ChatGPT’s abilities seemed easily transferrable to this exercise.

ChatGPT’s user dependability has also created a new competency in “prompt engineering.” The skills required to work with ChatGPT are not necessarily innate to all medical educators or tech-savvy users. The ease of using the tool is dependent on the technological skills and varied approaches of the individuals. In this study, one faculty with limited technological skills found the tool to be difficult to use and a burden trying to figure out how to get answers. However, another faculty with advanced technological skills found the tool to be easy to use and felt that it improved time efficiency. The user variation was not only evident in this focus group but has also spurred authors to create guides for medical professionals in their use of ChatGPT [11,12]. Initial prompts entered into ChatGPT for each case varied from 19 to 303 words, ranging from a list of detailed instructions and objectives to a simplified list of case components. One faculty noted that ChatGPT would “stall” after some of their prompts, and another felt that prompts needed to be “smaller,” otherwise the responses would be too superficial. Comparing the different transcripts, the output from ChatGPT did not seem to be noticeably different depending on the length of the prompts. Instead, authors have advised users to refine their prompt by thinking about its elements, such as instruction, context, input data, and output indicators [11]. Still, others and the users in this study have noted the importance of experimenting with different prompts and keeping directions simple [12]. As this new technology advances, so will its users, in order for its use in medical education to be successful. Medical schools will need to consider hiring experts in this field to monitor, teach, and continue navigating the advancements.

Concerns

As a large language model, ChatGPT “learns” from large datasets with additional fine-tuning of its programming and human feedback; however, it is not entirely clear what data ChatGPT has access to, and it continues to provide mixed results in medical practice [13]. Meanwhile, as it continues to “learn” from unverified sources of information and becomes more widely used, it begs the question of whether or not it will become its own echo chamber by referencing work that it has already created. For example, in the development of the cases, ChatGPT seemed to create very similar cases for each chief complaint, like chest pain. The cases appeared to be standardized in their formulation and were structured toward the most common diagnoses like acute coronary syndrome, consistently using descriptors like “crushing” or “pressure-like” chest pain. While the similarities between cases are due in part to similar prompts from the users, it seems clear that ChatGPT is unable to be “spontaneously creative” in this regard. Its dependence on the user’s prompts also carries forward numerous types of cognitive biases, like conscious or unconscious, anchoring or confirmation, etc. In medical education, students are able to reflect upon and learn from their own biases, whereas ChatGPT has the potential to repeat or echo the same sentiments over and over, diminishing diversity of thought.

When ChatGPT does stray from expectations, it seems to do so recklessly, changing the context of the conversation, confusing case discussion with actual patient care, or stubbornly providing incomplete or inaccurate information, frequently referred to as “hallucinations” or “fact fabrication” [4,9,13]. Many ChatGPT users have reported unusual interactions with the program, imbuing it with anthropomorphic qualities and creating feelings of discomfort and unrest [14]. The potential harm or impact to a medical student if ChatGPT is wrong, inappropriate, or possibly even manipulative can be extremely detrimental to the educational process. Heavy reliance on or integration of ChatGPT in formalized medical education sends an institutional message of validation of ChatGPT’s use. This should not be taken lightly given the potential for ChatGPT to be medically inaccurate and overly relied upon, particularly if ChatGPT’s language centers are coming from unverified sources and restricted from trusted sources such as PubMed or Cochrane [9,15].

Limitations

While this study highlights some of the pros and cons of using ChatGPT in the creation of medical cases used in education, there are a few limitations to consider. AI is a rapidly evolving technology, and since this study was completed, there have been many advances to the platform and multiple competing companies advancing the field. Qualitative studies also serve a great purpose in the discovery of ideas and concepts but do not provide statistical power. In this study, the authors cannot assess how common these strengths or shortcomings occur, making the results less generalizable. In a similar vein, while the faculty in this study were diverse in their experience with ChatGPT, they were all experienced educators at the same institution, and others using ChatGPT in medical education may have a very different experience or goal.

## Conclusions

ChatGPT is a tool currently used in medical education and has no limits to its capability. There is value to using ChatGPT to develop curricular content, especially for clinical reasoning cases, but it needs to be comprehensively reviewed and verified. Ultimately, ChatGPT was able to help create a baseline case that authors can heavily edit to meet the needs of the curriculum. To efficiently utilize the tool, educators will need to develop a framework that can be easily translatable into simple prompts that ChatGPT can understand. Future studies and work with ChatGPT will need to take a hard look at its capacity to not propagate biases and be cognizant of potential harms and misinformation.
